# Association between Several Clinical and Radiological Determinants with Long-Term Clinical Progression and Good Prognosis of Lower Limb Osteoarthritis

**DOI:** 10.1371/journal.pone.0025426

**Published:** 2011-10-21

**Authors:** Erlangga Yusuf, Jessica Bijsterbosch, P. Eline Slagboom, Herman M. Kroon, Frits R. Rosendaal, Tom W. J. Huizinga, Margreet Kloppenburg

**Affiliations:** 1 Department of Rheumatology, Leiden University Medical Center, Leiden, The Netherlands; 2 Department of Molecular Epidemiology, Leiden University Medical Center, Leiden, The Netherlands; 3 Department of Radiology, Leiden University Medical Center, Leiden, The Netherlands; 4 Department of Clinical Epidemiology, Leiden University Medical Center, Leiden, The Netherlands; Universidad Peruana Cayetano Heredia, Peru

## Abstract

**Objective:**

To investigate the factors associated with clinical progression and good prognosis in patients with lower limb osteoarthritis (OA).

**Methods:**

Cohort study of 145 patients with OA in either knee, hip or both. Progression was defined as 1) new joint prosthesis or 2) increase in WOMAC pain or function score during 6-years follow-up above pre-defined thresholds. Patients without progression with decrease in WOMAC pain or function score lower than pre-defined thresholds were categorized as good prognosis. Relative risks (RRs) for progression and good prognosis with 95% confidence interval (95% CI) were calculated by comparing the highest tertile or category to the lowest tertile, for baseline determinants (age, sex, BMI, WOMAC pain and function scores, pain on physical examination, total range of motion (tROM), osteophytes and joint space narrowing (JSN) scores), and for worsening in WOMAC pain and function score in 1-year. Adjustments were performed for age, sex, and BMI.

**Results:**

Follow-up was completed by 117 patients (81%, median age 60 years, 84% female); 62 (53%) and 31 patients (26%) showed progression and good prognosis, respectively. These following determinants were associated with progression: pain on physical examination (RR 1.2 (1.0 to 1.5)); tROM (1.4 (1.1 to 1.6); worsening in WOMAC pain (1.9 (1.2 to 2.3)); worsening in WOMAC function (2.4 (1.7 to 2.6)); osteophytes 1.5 (1.0 to 1.8); and JSN scores (2.3 (1.5 to 2.7)). Worsening in WOMAC pain (0.1 (0.1 to 0.8)) and function score (0.1 (0.1 to 0.7)), were negatively associated with good prognosis.

**Conclusion:**

Worsening of self-reported pain and function in one year, limited tROM and higher osteophytes and JSN scores were associated with clinical progression. Worsening in WOMAC pain and function score in 1- year were associated with lower risk to have good prognosis. These findings help to inform patients with regard to their OA prognosis.

## Introduction

Osteoarthritis (OA) of the lower limbs accounts for problems in performing lower extremities tasks such as walking and stair climbing.[1] Some of the patients with lower limb OA show progression of their OA with some progressing to total joint failure needing joint replacement.[2] Knowing those who will progress is important because it will improve patient information on the prognosis of OA.

Several studies have investigated determinants of the progression of knee and hip OA [3]–[5] and several remarks could be made on these studies. Firstly, none of the studies investigated knee and hip together. Investigating knee and hip separately is easy to understand but it does not reflect the clinical practice. In more than 30% of knee OA patients, hip OA is present at the same time [6] and up to 78% of patients have bilateral OA in knees or hips.[7] Concomitant presence of OA in lower limb joints will affect the experience of pain and influence disability in all lower limb joints. Arguably, it is difficult for a patient to allocate complaints to a particular knee or hip joint. The questionnaires used in OA, such as Western Ontario and McMaster Universities Osteoarthritis Index (WOMAC) asked questions on daily life activities such as climbing the stairs, where knee and hip joints are simultaneously needed.[8] Secondly, in most studies, progression was defined as joint deterioration on a radiograph while from the patient's perspective clinical progression is more important.[2], [9] Thirdly, almost exclusively baseline determinants of progression were investigated. However, OA patients are included in cohort studies at varying stages of the OA disease course, which make changes in determinants over a short time period of interest as prognostic factors on the long term.

Clinical progression is relevant for patients, but it is difficult to define. Probably this is one of the reasons why data on clinical progression are lacking compared to data on radiological progression. At this moment, there is no consensus on how to define clinical progression of knee and hip OA progression.[10], [11] Obviously, total joint replacement should be considered as OA disease progression. However, not all patients with worsening of their OA will receive joint replacement because of factors such as patient's comorbidity and surgeon's preference. Self-reported pain or disability could be used to define clinical progression, yet at present no standardized ‘cut-off’ for progression on self-reported outcome measures exists.

To deal with the abovementioned issues, we propose in the present study a composite outcome which combines total joint replacement and increase in self-reported pain and function during 6-years follow-up above a clinically relevant cut-off [8] as clinical progression. We sought to identify determinants associated with clinical progression and determinants associated with good prognosis of lower limb OA (knee and hip OA together). We assessed baseline determinants and determinants which were measured repeatedly over time.

## Materials and Methods

### Study design and patient population

This study is part of the Genetic ARthrosis and Progression (GARP) study, a cohort study aimed at identifying determinants of OA susceptibility and progression.[12] In this cohort, 192 Caucasian sib-pairs (384 patients), aged 40 to 70 years were included. To be included, patient should have symptomatic OA at multiple joint sites in the hands or OA in two or more of the following joint sites: hand, spine (cervical or lumbar), knee, or hip. Patients were recruited from the rheumatologic, orthopedic and general practice clinics around Leiden, The Netherlands. Patients with secondary OA, familial syndromes with a clear Mendelian inheritance, and a shortened life expectancy (<1 yr) were excluded. Patients underwent baseline assessment between August 2000 and March 2003 and filled-in questionnaires one year after this baseline visit. From April 2007 to June 2008 patients who consented for a follow-up evaluation (mean follow-up 6.1 years (range 5.1–7.5 years) were assessed.

To be eligible for the present study, patients needed to have OA in either knee or hip, or both. Knee OA was defined according to American College of Rheumatology (ACR) criteria as pain or stiffness in the knee on most days of the prior month and the presence of osteophytes in the tibiofemoral joints.[13] Hip OA was also defined according to ACR criteria as pain or stiffness in the groin and hip region on most days of prior month together with femoral or acetabular osteophytes or joint space narrowing on the radiograph.[14] There were 168 patients with knee or hip OA in the GARP cohort. Of these patients, 23 patients with prosthesis at baseline were excluded leaving 145 patients eligible for the follow-up. Patients with prosthesis at baseline were excluded because these patients could be considered as already having progressive disease at baseline and because having first prosthesis could influence the decision in having another prosthesis (confounder). This study was approved by the Medical Ethics Committee of the Leiden University Medical Center. Written informed consents form were obtained from all participants.

### Clinical assessment

Demographic data at baseline were recorded using standardized questionnaires. Self-reported pain (five items) and functional limitations (17 items) were evaluated by using the Dutch version of the WOMAC in 100 mm visual analogue scale format at baseline, at 1-year and at 6-year follow-up. It considered both knees and hips in the last 48 hours. Total scores on the pain and function subscales range from 0–100, higher scores indicated worse outcome.

Physical health at baseline was assessed with the summary component scales for physical health (PCS) of the Dutch validated Medical Outcomes Study Short Form-36 (SF-36) derived from norm based data from the Dutch population (mean 50, standard deviation (SD) 10). [15], [16] Higher scores indicate better physical health.

Physical examinations were performed at baseline. Pain on passive movement of the knee and hip joint was assessed using the modified articular index described by Doyle et al [17] (range 0 to 3; 0: no pain, 1: patient expressed tenderness, 2: patient expressed tenderness and winced, 3: patient expressed tenderness, winced and withdrew the joint). The total pain score ranged from 0 to 12. Flexion and extension of the knee and flexion and endorotation of the hip were measured using a goniometer and summed up as total range of motion (tROM).

### Radiographs

Radiographs of the knees (posterior-anterior (PA); weight-bearing, non-fluoroscopic fixed-flexion protocol) and hips (PA; weight-bearing) at baseline were taken by a single experienced radiographer using a standard protocol with a fixed film focus distance (1.30 m). These analogue films were digitized using a film digitizer at a resolution corresponding to a pixel size of 100 mu. Using the OARSI atlas [18], two readers (EY, JB) scored the radiographs by consensus opinion. Osteophytes were graded 0 to 3 in the hip, on the medial and lateral femur and in the medial and lateral tibia. Joint space narrowing (JSN) was graded 0 to 3 in the hip, and in medial and lateral tibiofemoral compartments of the knees. Total scores for osteophytes ranged from 0–24 in the knees and 0–6 in the hips. Total scores for JSN ranged from 0–12 in the knees and 0–6 in the hips. Intra-reader reproducibility based on 25 randomly selected pairs of radiographs was excellent, with intra-class correlation coefficient (ICC) of 0.99 for osteophytes and 0.98 for JSN.

### Definition of progression and good prognosis

Clinical progression was defined as: 1) the acquirement of joint replacement during follow-up or 2) an increase in self-reported (WOMAC) pain or function from baseline to 6-years follow-up above the predefined MPCI (minimum perceptible clinical improvement). The joint replacement should be due to OA and not due to other forms of arthritis or trauma. MPCI was originally developed as threshold value to define treatment response in OA. The threshold values were 9.7 for WOMAC pain and 9.3 for WOMAC function.[8]

These threshold values with negative sign, were used to define good prognosis. Patients without progression who had decrease in WOMAC pain or function score in 6-years lower than −9.7 or −9.3, respectively, were defined as having good prognosis.

### Statistical analysis

Data were analyzed using PASW Statistics 17 (SPSS Inc., Chicago, Ill, USA). Mean changes (SD and 95% confidence interval (95% CI)) for WOMAC pain and function, PCS and pain on examination scores were calculated by subtracting baseline scores from follow-up scores. Mean changes of scores with the 95% CI that did not cross 0 was considered as significant. The self-reported pain and function change scores of every patient were plotted in cumulative probability plot.

Determinants of clinical progression were assessed using logistic regression analysis. We assessed the following baseline determinants age, sex, BMI, WOMAC pain and function scores, pain on physical examination, total range of motion (tROM) and radiographic scores. We also assessed the determinants worsening in WOMAC pain and function score in 1-year.

The following baseline determinants were categorized in tertiles: BMI, WOMAC pain and function, tROM, osteophytes, and JSN. Also categorized in tertiles were worsening in WOMAC pain and function in 1-year. Pain on physical examination was categorized into presence or absence of pain. In the logistic regression analysis, the odds ratios (ORs) were calculated by using the lowest category or the lowest tertile as reference except for tROM where the highest tertile was used as reference. The ORs were transformed to risk ratio (RRs) using the approximation formula of Zhang because ORs of common outcomes in a fixed cohort are not a good approximation of RRs.[19] Since the population of this study consists of sib pairs, intrafamily effect were taken into account by computing robust standard errors using Stata version 8 (Stata, College Station, Tx, USA). In the analyses, adjustments were made for age, sex, and BMI. A significant determinant of progression was defined as a determinant that the 95% CI of its RR did not cross 1.

The significant determinants were included in a multivariate model to investigate whether these determinants could independently predict the clinical progression. To get an impression on how good these determinants predict clinical progression when they presented together, the R^2^ of this model was determined. Additionally, to investigate the discriminative ability of the multivariate model, we fitted a receiver operating characteristics curve (ROC) and calculated the area under the curve (AUC). We compared the predicted risk of progression with the observed clinical progression and good prognosis with the observed clinical progression and good prognosis.

## Results

### Population description

Of 145 patients eligible for the follow-up, 117 (81%) gave consent for follow-up assessment. The reasons for non-consent were: no interest in the follow-up study (n = 8), unavailability of transport (n = 8) health problems not associated with OA (n = 4), emigration (n = 1), and unknown (n = 2). Five patients died during follow-up.

Baseline characteristics of patients with and without follow-up and excluded patients due to joint prosthesis at baseline are presented in Table 1. No difference was found between baseline characteristics of patients with and without follow-up (Table 1).

**Table 1:** Baseline characteristics of 168 patients with knee and/or hip OA stratified by availability of follow-up.

	*Follow-up (n = 117)*	*No follow-up (n = 28)*	*Joint prosthesis at baseline (n = 23)*
Age, yrs, median (IQR)			
	60 (55–66)	62 (53–58)	64 (61–68)
Female, no (%)			
	98 (84)	24 (74)	13 (72)
BMI, kg/m^2^, mean (range)			
	28.0 (20 to 47)	27.3 (20 to 38)	29.3 (22 to 43)
Patients with OA, no (%)			
Knee	74 (63)	18 (55)	3 (17)
Hip	31 (27)	6 (18)	6 (33)
Knee and hip	11 (10)	9 (27)	9 (50)
Total range of motion, °, mean (range)			
	258 (133 to 389)	257 (219 to 441)	251 (48 to 360)
Knee flexion	86 (1 to 155)	86 (0 to 155)	85 (16 to 135)
Knee extension.	−4 (−30 to 10)	−3 (−30 to 16)	−2 (−15 to 16)
Hip flexion	134 (100 to 176)	134 (8 to 166)	133 (8 to 175)
Hip extension	41 (0 to 80)	39 (0 to 80)	26 (8 to 49)
Joint prosthesis, no.			
	n/a	n/a	23
Hip			16
Knee			6
Knee and hip			1
Presence of pain on physical examination, no (%)*			
	85 (73)	20 (71)	17 (74)
Hip	30 (26)	9 (32)	14 (61)
Knee	64 (55)	16 (57)	11 (48)

*Patients may have OA at multiple joints at one time and can have pain in the knee and hip joint simultaneously. Abbreviation: IQR: interquartile range; BMI: Body Mass Index.

### Clinical course of lower limb osteoarthritis

The mean changes (95%CI) of self-reported (WOMAC) pain and function scores of all patients were −2.6 (−8.9 to 3.7) and 0.5 (−5.9 to 6.9), respectively (Table 2).

**Table 2:** Mean (standard deviation (SD)) baseline, follow-up (FU), and change scores on self-reported pain and function (WOMAC), physical health (PCS), and pain on physical examination (PE) for the total population and sub-groups.

		*Baseline*	*Follow-up*	*Change (95% CI)*
All patients (n = 117)	WOMAC pain	36.2 (23.5)	33.6 (25.7)	−2.6 (−8.9 to 3.7)
	WOMAC function	33.1 (24.3)	33.6 (24.8)	0.5 (−5.9 to 6.9)
	PCS	41.8 (9.8)‡	42.0 (10.1)‡	0.2 (−2.4 to 2.8)
	Pain on PE	1.7 (1.7)	2.4 (2.4)	0.7 (0.2 to 1.2)‡
Patients receiving prosthesis during FU (n = 36)	WOMAC pain	36.5 (18.2)	28.0 (21.0)	−8.5 (−17.8 to−0.1)‡
	WOMAC function	32.4 (20.1)	30.0 (20.6)	−2.4 (−12.0 to 7.2)
	PCS	40.8 (9.1)‡	40.7 (10.0)‡	−0.1 (−4.6 to 4.4)
	Pain on PE	1.8 (1.6)	2.8 (3.1)	1.0 (−0.2 to 2.2)
Patient not receiving prosthesis during FU (n = 81)	WOMAC pain	36.1 (25.6)	36.0 (27.2)	−0.1 (−8.3 to 8.1)
	WOMAC function	33.4 (26.1)	35.3 (26.4)	1.9 (−6.3 to 10.1)
	PCS	42.3 (10.1)‡	42.6 (10.0)‡	0.3 (−2.8 to 3.4)
	Pain on PE	1.7 (1.8)	2.3 (2.1)	0.6 (−0.01 to 1.2)

‡ : statistically significant; the significance of physical health summary were tested by comparing the study sample with the norm based population (mean = 50, SD = 10).

During follow-up, 36 patients (31%) received at least one joint replacement; 15 for the hip, 16 for the knee, and five for both knee and hip. In these patients with new joint replacements, the mean WOMAC pain score (95% CI) decreased over the six years of follow-up (−8.5 (−17.8 to −0.1). In the patients without new prosthesis (n = 81), WOMAC pain and WOMAC function scores did not change significantly over time: −0.1 (−8.3 to 8.1) and 1.9 (−6.3 to 10.1), respectively.

**Figure 1:**
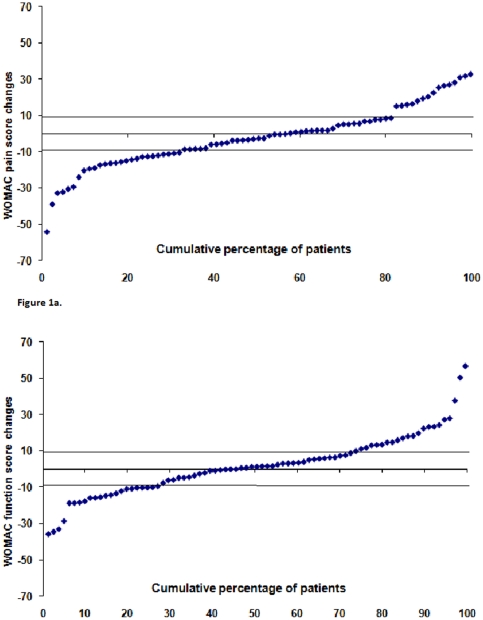
Cumulative probability plot of Western Ontario and McMaster Universities (WOMAC) scores change of patients without prosthesis during follow-up (n = 81) for WOMAC pain scores change (above) and WOMAC function scores change (below). The horizontal line above is the line set at minimal perceptible clinical improvement (MPCI) score which is used as the cut-off to define progression and the horizontal line below is the line set to define good prognosis.

Cumulative probability plots show the variation in natural course of self-reported pain and function in the sub-group of patients without prosthesis (n = 81) (Figures 1a and 1b). Fifteen and 22 patients showed progression of WOMAC pain and WOMAC function based on changes above the MCPI, respectively. In total, 26 patients (19.7%) showed clinical deterioration. Together with the 36 patients receiving joint replacement during follow-up, 62 of 117 patients (53.0%) showed clinical progression. Thirty-one patients showed good prognosis, based on change in WOMAC pain or WOMAC function score change lower than −9.7 (n = 23) or −9.3 (n = 22), respectively.

In the total study sample, in the subgroup of patients with new prosthesis, and in patients without new prosthesis, physical health summary measures using SF-36 did not change during follow-up (Table 2). Compared to the general population (mean of 50 with SD of 10), physical health of lower limb OA patients was consistently shown to be worse at baseline and follow-up.

Pain during physical examination was worsened in the total population (Table 2). In the sub-group with new prosthesis, pain did not worsen.

### Determinants of clinical progression of lower limb osteoarthritis

Determinants of clinical progression of lower limb OA are shown in Table 3. Age, female sex, and BMI, were not associated with clinical progression. Worsening of WOMAC pain and function scores in the first year were associated with 6-year progression while WOMAC pain and function score at baseline were not. Subjects in the highest tertile of WOMAC pain and function worsening in 1 year had a RR (95% CI) of 1.9 (1.2 to 2.3) and 2.4 (1.7 to 2.7), respectively, for clinical progression. The presence of pain on physical examination at baseline was associated with clinical progression (1.2 (1.0 to 1.5)). Patients in the lowest tertile of tROM had a higher risk for clinical progression RRs of 1.4 (1.1 to 1.6)). Osteophytes and JSN at baseline were associated with clinical progression, RR for being in the highest tertile of osteophytes and JSN scores were 1.5 (1.0 to 1.8) and 2.3 (1.5 to 2.6), respectively. In a multivariate regression model, WOMAC function worsening in 1 year, limited t ROM, and JSN scores were found as independent determinants of clinical progression (Table 3). With these variables, explained variance (R^2^) was 48.6%. The AUCs of the ROC curves were 0.85 (95% CI 0.76 to 0.94).

**Table 3:** Determinants for clinical progression over 6 years of lower limb osteoarthritis.

*Determinant*	*Number of patients*		*Risk ratio (95% CI) 1*	*Risk ratio (95% CI) 2*
	*Pro-gression (%)*	*No pro-gression (%)*		
Age>60 years	59 (50)	50 (43)	1.0 (0.9 to 1.1)	na
Female sex	48 (41)	50 (43)	0.6 (0.3 to 1.0)	na
Body mass index (kg/m^2^)				
<25.5	19 (16)	20 (17)	1	na
25.5 to 29.1	16 (14)	21 (18)	0.9 (0.5 to 1.2)	
>29.1	27 (23)	14 (12)	1.3 (0.9 to 1.7)	
WOMAC pain scores				
0 to 23.2	21 (18)	18 (15)	1	na
23.2 to 45.9	20 (17)	18 (15)	0.9 (0.5 to 1.3)	
>45.9	21 (18)	19 (16)	1.1 (0.7 to 1.4)	
WOMAC function scores				
0 to 18.0	20 (17)	20 (17)	1	na
18.0 to 40.9	22 (19)	16 (14)	1.2 (0.7 to 1.6)	
>40.9	20 (17)	19 (16)	1.1 (0.7 to 1.5)	
Change in WOMAC pain score in 1 year				
<−3.3	10 (9)	16 (14)	1	na
−3.3 to 10.1	15 (13)	11 (9)	1.6 (0.8 to 2.2)	
>10.1	17 (15)	9 (8)	1.9 (1.2 to 2.3)‡	
Change in WOMAC function score in 1 year				
<−1.4	9 (8)	17 (15)	1	1
−1.4 to 6.7	13 (11)	14 (12)	1.5 (0.9 to 2.7)	1.9 (0.9 to 2.6)
>6.7	20 (17)	5 (4)	2.4 (1.7 to 2.7)‡	2.3 (1.2 to 2.8)‡
Pain on physical examination	44 (38)	13 (11)	1.2 (1.0 to 1.5)‡	1.2 (0.8 to 1.2)
Total range of motion (°)				
>554	14 (12)	25 (21)	1	1
522 to 554	25 (21)	14 (12)	1.4 (1.01 to 1.7)	1.2 (0.9 to 1.2)
<522	23 (20)	16 (14)	1.4 (1.1 to 1.6)‡	1.2 (1.03 to 1.3)‡
Osteophyte scores				
1	19 (16)	28 (24)	1	na
2 to 4	19 (16)	10 (9)	1.4 (1.0 to 3.8)‡	
>4	11 (9)	8 (7)	1.5 (1.0 to 1.8)‡	
JSN scores				
1	19 (16)	32 (27)	1	1
2 to 4	16 (14)	12 (10)	1.5 (0.9 to 2.1)	1.6 (0.7 to 2.4)
>4	14 (12)	2 (2)	2.3 (1.5 to 2.6)‡	2.4 (1.9 to 2.7)‡

^*1*^except for determinants age, sex and BMI themselves, adjustment was made for age, sex and BMI.

^*2*^multivariate model using a backward selection (R^2^ = 48.6%). The independent variables with univariate associations with a p-value ≤0.10 were included.

Both models are calculated using approximation formula of Zhang.[19].

‡ : statistically significant.

Abbreviations: WOMAC: Western Ontario and McMaster Universities, JSN: joint space narrowing, na: not applicable.

### Determinants of good prognosis of lower limb osteoarthritis

Worsening in WOMAC pain and function score in 1 year were negatively associated with good prognosis, i.e. patients in highest tertile of 1-year increase in WOMAC pain and function scores had lower risk to have good prognosis. (Table 4). Patients in the highest tertile of worsening of WOMAC pain and function in 1 year, had RR of 0.1 (95% CI 0.1 to 0.8) and 0.1 (0.1 to 0.7), respectively to have good prognosis of their lower limb OA compared to patients with WOMAC pain and function change in the lowest tertile. When these significant determinants were analyzed in one model, only worsening in WOMAC function in 1- year was negatively associated with good prognosis. The R^2^ of this model was 43.3% and the AUCs of the ROC curves were 0.78 (0.68 to 0.89).

**Table 4:** Determinants of good prognosis of lower limb osteoarthritis over 6 years.

*Determinant*	*Number of patients*		*Risk ratio (95% CI) 1*	*Risk ratio (95% CI) 2*
	*Good prognosis (%)*	*Without good prognosis (%)*		
Age>60 years	28 (24)	3 (3)	1.0 (0.7 to 1.0)	na
Female sex	29 (25)	68 (58)	2.8 (0.8 to 6.3)	na
Body mass index (kg/m^2^)				
<25.5	14 (12)	25 (21)	1	na
25.5 to 29.1	12 (10)	25 (21)	0.9 (0.4 to 1.6)	
>29.1	5 (4)	35 (30)	0.3 (0.1 to 0.9)	
WOMAC pain scores				
0 to 18.0	4 (4)	34 (29)	1	na
18.0 to 45.9	14 (12)	24 (20)	2.7 (0.7 to 3.6)	
>40.9	13 (11)	27 (23)	2.2 (0.7 to 3.8)	
WOMAC function scores				
0 to 18.0	6 (5)	34 (29)	1	na
18.0 to 40.9	13 (11)	24 (20)	2.5 (0.1 to 4.5)	
>40.9	12 (10)	27 (23)	1.9 (0.7 to 3.8)	
Change in WOMAC pain score in 1 year				
<−3.3	14 (12)	12 (10)	1	na
−3.3 to 10.1	5 (4)	21 (18)	0.3 (0.1 to 0.6)‡	0.6 (0.1 to 1.3)
>10.1	3 (3)	23 (20)	0.1 (0.1 to 0.8)‡	0.5 (0.1 to 1.1)
Change in WOMAC function score in 1 year				
<−1.4	15 (13)	11 (9)	1	1
−1.4 to 6.7	5 (4)	22 (19)	0.3 (0.1 to 0.7)‡	0.3 (0.1 to 0.8)‡
>6.7	2 (2)	23 (18)	0.1 (0.1 to 0.7)‡	0.2 (0.1 to 0.8)‡
Pain on physical examination	20 (17)	11 (9)	0.9 (0.6 to 1.1)	na
Total range of motion (°)				
>554	12 (10)	27 (23)	1	na
522 to 554	10 (9)	28 (24)	0.8 (0.3 to 1.7)	
<522	9 (8)	30 (26)	0.9 (0.4 to 1.8)	
Osteophyte scores				
1	17 (15)	30 (26)	1	na
2 to 4	6 (5)	23 (20)	0.6 (0.2 to 1.2)	
>4	4 (3)	15 (13)	0.5 (0.2 to 1.3)	
JSN scores				
1	18 (15)	33 (28)	1	na
2 to 4	7 (6)	21 (18)	0.7 (0.3 to 1.4)	
>4	2 (2)	14 (12)	0.4 (0.1 to 1.4)	

1 except for determinants age, sex and BMI themselves, adjustment was made for age, sex and BMI.

2 multivariate model using a backward selection (R^2^ = 43.3%). The independent variables with univariate associations with a p-value ≤0.10 were included.

Both models are calculated using approximation formula of Zhang.[19].

‡ : statistically significant.

Abbreviations: WOMAC: Western Ontario and McMaster Universities, JSN: joint space narrowing, na: not applicable.

## Discussion

To our knowledge, the present study is the first which investigated determinants of clinical progression of knee and hip together. Clinical outcome is chosen because it is essential to patients. Clinical progression was present in 53% of patients; 33% by receiving joint prosthesis and 20% by deteriorating of self-reported pain or function.

Self-reported pain improved over 6 years in patients who received prostheses. Self-reported function did not change over 6 years regardless of joint replacement. The combination of WOMAC function changes in 1 year, limited tROM, and JSN scores provided the best explanation of variation in clinical progression of lower limb OA. Worsening WOMAC pain and function in 1 year were negatively associated with good prognosis. Patients in the highest tertile of worsening in WOMAC pain and WOMAC function in 1-year had 90% less chance to have good prognosis of their lower limb OA compared to patients with pain and function change in the lowest tertile.

The proportion of the study sample showing clinical progression in our study is comparable to results from the Bristol ‘OA 500 study’. In that descriptive study, where the majority of the study population was also female, clinical change was reported by the patients as: better, same, and worse. They found that 63% and 54% of the patients reported worsening in overall condition for the knee and hip respectively, after 8 years follow-up.[9] In the present study, self-reported pain and function for the whole group did not change in 6 years. This can be explained by the variation in progression between individuals as depicted in the cumulative probability plots (Figure 1a and 1b). Although some patients remained stable and even reported improvement, a considerable proportion of patients reported more pain and worse function. As a result the mean change is small. As expected in the sub-group of patients receiving joint prosthesis during follow-up, self-reported pain improved over 6 years, however, self-reported function did not. These results are consistent with the notion that joint replacement is an effective treatment for pain in lower limb OA. However, it seems that joint replacement cannot replace the function of the natural joint. Our results showed some parallels with a recent study by Nilsdotter et al.[20] They showed that patients had high preoperative expectations concerning reduction of pain and function but one year after knee replacement only the expectation regarding reduction of pain was fulfilled.

While self-reported pain at baseline was not associated with clinical progression, rapid deterioration in self-reported pain and function in the first year (even after correction for WOMAC scores at baseline that could confound the association) was associated with higher risk of progression over 6 years. This has not been studied before in OA, but it is in accordance with studies in rheumatoid arthritis (RA): worsening in self-reported disability measured with the health assessment questionnaire was a predictor for severe RA outcomes on the long term.[21] Interestingly, worsening in WOMAC pain and function score in 1-year were negatively associated with good prognosis. The consequence of these findings is that by following lower limb OA patients for 1 year, doctors can inform the patients about the progression of the OA in the long term. Therefore, it might advisable that doctors see their patients again 1-year after the first visit. It will be also interesting to investigate in a clinical trial whether modification of self reported pain or function one year after the presentation by means of physical therapy or better pain medication could stop the clinical progression of OA.

Pain on physical examination at baseline was associated with clinical progression. It was also the only pain variable that deteriorated over time. This observation reflects that pain as reported by the patient differs from pain on passive movement as found during physical examination as shown previously.[22]

Limited tROM (RR 1.4, 95%CI 1.1 to 1.6) and presence of pain on physical examination at baseline (RR 1.2, 95%CI 1.0 to 1.5) probably reflected the structural damage and might be used as a surrogate for osteophyte and JSN scores. In a recent EULAR recommendation for the diagnosis of knee OA, limited movement was indeed proposed as one of the clinical signs needed to make the diagnosis, probably because it was associated with radiological OA.[23]

Osteophytes and JSN scores were also identified as determinants of lower limb OA progression. Our findings support the findings of Lane and colleague, that osteophyte, JSN together with subchondral bone sclerosis were associated with hip OA progression.[4]

We showed that the WOMAC function changes in 1 year, limited tROM and higher JSN scores were independently significant determinants of clinical progression of lower limb OA. Although the main aim of this paper was to identify the determinants that were associated with clinical progression and not to build a prognostic model, we tried to get an impression on how good these determinants in predicting clinical progression when they were present together. We also tested the discriminative ability of this model to get an indication on how good the presence of these determinants predicts the clinical progression of lower OA. Their cumulative presence provided a very good explanation of variation in clinical progression, as shown with R^2^ of 48.6%. The AUCs of the ROC curves of 0.80 also indicates a reasonable discriminative ability. This means that performing assessment on these three determinants in clinical practice will help clinician much in predicting the progression of lower limb OA and therefore give better patient information.

Roos et al showed that female sex was associated with worsening in self-reported pain and function and that older age and higher BMI were associated with worsening in function assessed on physical examination. On the other hand, we found no associations between demographic determinants and clinical progression.[5] Determinants for incidence are often failed to be identified as determinant of progression. The failure in finding determinants for progression is a common phenomenon that might be caused by methodological problem in studies restricted to subjects with existing disease.[24] Unfortunately, no method is yet available to overcome this problem. Another possible explanation in the difference in our results and results from Roos et al is the difference in patient population. The population in the study of Roos was a mix of knee OA patients and participants who underwent menisectomy in the past.

Our study sample that consists of selected sib-pairs with OA at multiple sites has strengths and limitations. Since generalized OA (GOA) population is associated with rapid OA progression [25], our study population is suitable to investigate OA progression within a relatively short period. However, the generalizability of our results in other population settings, especially to general practice clinics is arguably limited and we could not investigate GOA as determinant for progression. Yet, if we compare the ‘severity of OA’ by taking the incidence of joint prosthesis, we did not see much difference in the incidence of joint prosthesis in our study sample and in a hospital based OA population which was not selected for GOA, for a comparable follow-up time.[9] It supports the observations in other patient populations that generalized OA is also common and it is important to bear in mind that OA is often present at multiple sites while only the most symptomatic sites draw attention.[9], [25] To leave out the familial effect, we have performed a correction for familial factors in analysis.

The choice of the composite outcome that is a combination of two outcomes: joint prosthesis and increase in WOMAC pain or function scores above MPCI rewards comments. The two outcomes might be different; increase in WOMAC scores above MPCI might not always results in joint prosthesis. Also, the use of MPCI in defining progression is arbitrary. It was originally created to indicate clinical improvement in trials.[8] However, since no clinical outcome regarding clinical progression of knee or hip or lower limb OA is available at this moment, our choice of outcome could be considered to be used in observational studies.

It should be noted that our study population consists mainly of female. OA is known to be more common in female. The phenomenon that female tend to be overrepresented in OA studies is well known, such as in the large Bristol ‘OA 500 study’ mentioned above.[9] In the present study, effort has been taken to adjust for this possible confounder.

In summary, over a period of 6 years, more than half of the patients showed progression of lower limb OA, based on total joint replacement or change in self-reported pain or function above the MPCI. Performing combination of clinical and radiological assessment in clinical practice could evaluate the sub-group of patients with progression of lower limb OA. These findings would help doctors in patient information regarding progression of lower limb OA.
